# Interpreting HIV diagnostic histories into infection time estimates: analytical framework and online tool

**DOI:** 10.1186/s12879-019-4543-9

**Published:** 2019-10-26

**Authors:** Eduard Grebe, Shelley N. Facente, Jeremy Bingham, Christopher D. Pilcher, Andrew Powrie, Jarryd Gerber, Gareth Priede, Trust Chibawara, Michael P. Busch, Gary Murphy, Reshma Kassanjee, Alex Welte, on behalf of the Consortium for the Evaluation and Performance of HIV Incidence Assays (CEPHIA), Alex Welte, Joseph Sempa, David Matten, Hilmarié Brand, Trust Chibawara, Gary Murphy, Jake Hall, Elaine Mckinney, Michael P. Busch, Eduard Grebe, Shelley Facente, Dylan Hampton, Sheila Keating, Mila Lebedeva, Christopher D. Pilcher, Kara Marson, Reshma Kassanjee, Oliver Laeyendecker, Thomas Quinn, David Burns, Susan Little, Anita Sands, Tim Hallett, Sherry Michele Owen, Bharat Parekh, Connie Sexton, Matthew Price, Anatoli Kamali, Lisa Loeb, Jeffrey Martin, Steven G. Deeks, Rebecca Hoh, Zelinda Bartolomei, Natalia Cerqueira, Breno Santos, Kellin Zabtoski, Rita de Cassia Alves Lira, Rosa Dea Sperhacke, Leonardo R. Motta, Machline Paganella, Esper Kallas, Helena Tomiyama, Claudia Tomiyama, Priscilla Costa, Maria A. Nunes, Gisele Reis, Mariana M. Sauer, Natalia Cerqueira, Zelinda Nakagawa, Lilian Ferrari, Ana P. Amaral, Karine Milani, Salim S. Abdool Karim, Quarraisha Abdool Karim, Thumbi Ndungu, Nelisile Majola, Natasha Samsunder, Denise Naniche, Inácio Mandomando, Eusebio V. Macete, Jorge Sanchez, Javier Lama, Ann Duerr, Maria R. Capobianchi, Barbara Suligoi, Susan Stramer, Phillip Williamson, Marion Vermeulen, Ester Sabino

**Affiliations:** 1 Vitalant Research Institute, 270 Masonic Avenue, San Francisco, CA 94118 USA; 2 DST-NRF Centre of Excellence in Epidemiological Modelling and Analysis (SACEMA), Stellenbosch University, 19 Jonkershoek Avenue, Stellenbosch, 7925 South Africa; 3 University of California San Francisco, 1001 Potrero Avenue, Room 5H22, San Francisco, CA 94110 USA; 4 Facente Consulting, Richmond, CA USA; 5 Implicit Design, Block B, North Building Black River Park, 2 Fir St, Observatory, Cape Town, 7925 South Africa; 6 Public Health England, 61 Colindale Avenue, London, NW9 5EQ UK; 7 Centre for Infectious Disease Epidemiology and Research, School of Public Health and Family Medicine, Faculty of Health Sciences, University of Cape Town, Observatory, Cape Town, 7925 South Africa

**Keywords:** HIV, Infection dating, Infection duration, Infection timing, Diagnostics, Diagnostic assays

## Abstract

**Background:**

It is frequently of epidemiological and/or clinical interest to estimate the date of HIV infection or time-since-infection of individuals. Yet, for over 15 years, the only widely-referenced infection dating algorithm that utilises diagnostic testing data to estimate time-since-infection has been the ‘Fiebig staging’ system. This defines a number of stages of early HIV infection through various standard combinations of contemporaneous discordant diagnostic results using tests of different sensitivity. To develop a new, more nuanced infection dating algorithm, we generalised the Fiebig approach to accommodate positive and negative diagnostic results generated on the same *or* different dates, and arbitrary current or future tests – as long as the test sensitivity is known. For this purpose, test sensitivity is the probability of a positive result as a function of time since infection.

**Methods:**

The present work outlines the analytical framework for infection date estimation using subject-level diagnostic testing histories, and data on test sensitivity. We introduce a publicly-available online HIV infection dating tool that implements this estimation method, bringing together 1) curatorship of HIV test performance data, and 2) infection date estimation functionality, to calculate plausible intervals within which infection likely became detectable for each individual. The midpoints of these intervals are interpreted as infection time ‘point estimates’ and referred to as Estimated Dates of Detectable Infection (EDDIs). The tool is designed for easy bulk processing of information (as may be appropriate for research studies) but can also be used for individual patients (such as in clinical practice).

**Results:**

In many settings, including most research studies, detailed diagnostic testing data are routinely recorded, and can provide reasonably precise estimates of the timing of HIV infection. We present a simple logic to the interpretation of diagnostic testing histories into infection time estimates, either as a point estimate (EDDI) or an interval (earliest plausible to latest plausible dates of detectable infection), along with a publicly-accessible online tool that supports wide application of this logic.

**Conclusions:**

This tool, available at https://tools.incidence-estimation.org/idt/, is readily updatable as test technology evolves, given the simple architecture of the system and its nature as an open source project.

## Background

For pathogenesis studies, diagnostic biomarker evaluation, and surveillance purposes, it is frequently of interest to estimate the HIV infection time of study subjects (i.e., the date of infection or time-since-infection). Ideally, a biomarker signature would provide reasonable direct estimates of an individual’s time-since-infection, but natural inter-subject variability of pathogenesis and disease progression makes this difficult. This work presents a general schema for utilising qualitative (i.e. positive/negative) diagnostic test results to estimate the time of HIV infection. Such estimates can be further refined by interpreting quantitative results on diagnostic or staging assays [1].

Most simply, nuanced infection dating applies to subjects who produce at least one negative test result and at least one positive test result (usually at a later time), taking into account that no test can detect infection immediately after infectious exposure. Hence, infection can at best be estimated to have occurred during an interval in the past, relative to the dates of the tests.

When a subject obtains discordant results, i.e. a negative and a positive test result on the same day, this typically manifests as positive results on ‘more sensitive’ tests than those on which the negative results were obtained. For high-performing diagnostic tests, such as are normal for HIV and other viral infections like hepatitis C, test sensitivity is best understood as the probability of identifying a positive case as a function of time since infection (which is conventionally summarised as merely the probability of correctly identifying a positive case).

For more than 15 years, the only widely-referenced infection dating algorithm using diagnostic test results to estimate time-since-infection has been the ‘Fiebig staging’ system [2]. This system defines a number of stages of early HIV infection through various standard combinations of discordant results using diagnostic tests of different sensitivity, with specimens from the same day. For example, Fiebig stage 1 is defined as exhibiting reactivity on a viral load assay, but not (yet) on a p24 antigen assay, and in the seminal 2003 paper was estimated to begin approximately 11 days after infection, with a mean duration of 5.0 days [2]. The particular tests used in these original calculations are largely no longer in use, nor commercially available. Others have used newer diagnostic assays to recalibrate the Fiebig stage mean duration estimates or define similar stages as an analogue to the Fiebig method [3, 4], though as tests evolve and proliferate, it becomes infeasible to calibrate all permutations of test discordancy.

Building from the Fiebig staging concept, we developed a new, more nuanced infection dating algorithm to meet the needs of the Consortium for the Evaluation and Performance of HIV Incidence Assays (CEPHIA) in support of the discovery, development and evaluation of biomarkers for recent infection [5–7]. The primary CEPHIA activity has been to develop various case definitions for ‘recent HIV infection’, with intended applicability mainly to HIV incidence surveillance rather than individual-level staging, although the latter application has also been explored [6, 8]. CEPHIA has been able to identify large numbers of well-characterized specimens and provide consistent conditions in which to conduct laboratory evaluations of several candidate incidence assays [7, 9]. A key challenge faced by CEPHIA was that specimens in the repository had been collected from numerous studies, each of which used different diagnostic algorithms, therefore capturing different information about the timing of HIV acquisition or seroconversion. To meet this challenge, we had to link specimens from thousands of study-patient interactions into a coherent and consistent infection dating scheme, which enabled interpretation of arbitrary diagnostic test results (as long as the performance of the tests in question were known). This general approach was first described in [5], but substantially refined in the present work.

In order to align diagnostic testing information across multiple sources, one needs a common reference event in a patient history – ideally, the time of an exposure that leads to infection. When dealing with actual patient data, however, we are usually constrained to estimate the time when a particular test (X) would have first detected the infection. We will call this the test(X)-specific *Date of Detectable Infection*, or DDI_X_.

The present work outlines the analytical framework for infection date estimation using ‘diagnostic testing histories’, and introduces a publicly-available online HIV infection dating tool that implements this estimation, bringing together 1) curatorship of HIV test performance data, and 2) infection date estimation functionality. It is readily updatable as test technology evolves, given the simple general architecture of the system and its nature as an open source project.

## Methods

### Generalised Fiebig-like staging

The fundamental feature of the Fiebig staging system [2] is that it identifies a naturally-occurring sequence of discordant diagnostic tests, which together indicate early clinical disease progression. The approximate duration of infection can be deduced by analysing the combination of specific assay results and assigning the appropriate “stage”.

As we demonstrate below, it is preferable to interpret *any* combination of diagnostic test results into an estimated duration of infection, if these tests have been independently benchmarked for diagnostic sensitivity (i.e. a median or mean duration of time from infection to detectability on that assay has been estimated). Unlike Fiebig staging, this more nuanced method allows both for incorporation of results from any available test, and from results of tests run on specimens taken on different days.

**Fig. 1:**
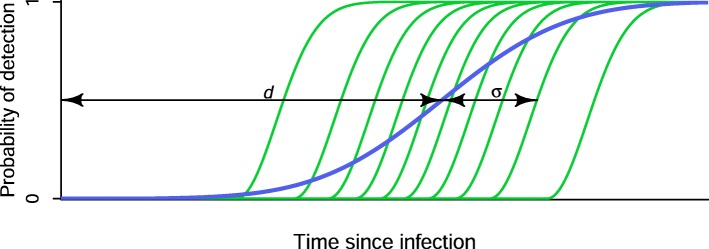
Diagnostic test sensitivity as a function of time since infection. The green curves show individual subject-level test sensitivities, and the blue curve shows the population-level average

In contrast to the usual statistical definition of ‘sensitivity’ as the proportion of ‘true positive’ specimens that produce a positive result, we summarise the population-level sensitivity of any particular diagnostic test into one or two ‘diagnostic delay’ parameters (*d* and *σ* in Fig. 1). Interpreted at the population level, a particular test’s sensitivity curve expresses the probability that a specimen obtained at some time *t* after infection will produce a positive result. The key features of a test’s sensitivity curve (represented by the blue curve in Fig. 1) are that:

- there is effectively no chance of detecting an infection immediately after exposure;
- after some time, the test will almost certainly detect an infection;
- there is a characteristic time range over which this function transitions from close to zero to close to one. This can be summarised as something very much like a mean or median and a standard deviation.

By far the most important parameter is an estimate of ‘*median diagnostic delay’*. In Fig. 1, this is the parameter *d*. If there were perfect test result conversion for all subjects (i.e. no assay ‘noise’), and no inter-subject variability, this would reduce the smoothly varying blue curve to a step function.

Various host and pathogen attributes, such as concurrent infections, age, pregnancy status, the particular viral genotype, post-infection factors, etc., affect the performance of a test for a particular individual. This determines a subject’s specific sensitivity curve, such as one of the green curves in Fig. 1, which capture the probability that specimens *from a particular subject* will produce a positive diagnostic result. Because assay results are themselves not perfectly reproducible even on the same individual, even these green curves do not transition step-like from zero to one, but rather have some more finite window of time over which they transition from close to zero to close to one.

To estimate individual infection times, then, one needs to obtain estimates of the median diagnostic delays and intersubject variability (i.e. the purple curve in Fig. 1) for all tests occurring in a data set, and then interpret each individual assay result as excluding segments of time during which infection was not possible, ultimately resulting in a final inferred interval of time during which infection likely occurred.

These calculations require that each individual has at least one negative test result and at least one positive test result. In the primitive case where there is precisely one of each, namely a negative result on a test with an expected diagnostic delay of *d*_1_ at *t*_1_ and a positive result on a test with an expected diagnostic delay of *d*_2_ at *t*_2_, then the interval is simply from (*t*_1_ − *d*_1_) to (*t*_2_ − *d*_2_). When there are multiple negative results on tests at ${t}_i^{\left(-\right)}$ each with a diagnostic delay ${d}_i^{\left(-\right)}$, and/or multiple positive results on tests at ${t}_j^{\left(+\right)}$ each with a diagnostic delay ${d}_j^{\left(+\right)}$, then each individual negative or positive test result provides a candidate earliest plausible and latest plausible date of infection. The most informative tests are the ones that most narrow the ‘infection window’ (i.e. result in the latest start and earliest end of the window). In this case, the point of first ‘detectability’ refers to the time when the probability of infection being detected by an assay first exceeds 0.5.

**Fig. 2:**
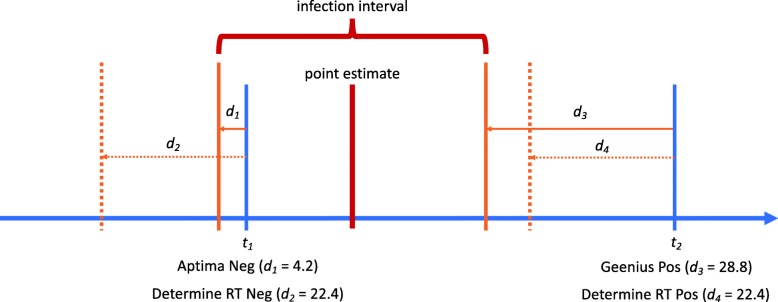
Example of infection time point estimate and interval obtained for a hypothetical subject who tested negative on the Aptima qualitative NAT assay and Determine rapid test at *t*_*1*_, and positive on the Geenius supplemental assay and Determine rapid test at *t*_*2*_

These remaining plausible ‘infection windows’ are usually summarised as intervals, the midpoint of which is naturally considered a ‘point estimate’ of the date of infection. Figure 2 illustrates the way this method works, on a particular (hypothetical) individual. Given two negative test results on one date and two positive test results on a later date, a plausible infection window can be estimated using the diagnostic delays of the assays in question (*d*_1_, *d*_2_, *d*_3_ and *d*_4_ in the figure). Note that it is the most sensitive negative test and the least sensitive positive test that proves most informative by excluding the greatest periods of time during which infection could not have occurred.

**Fig. 3:**
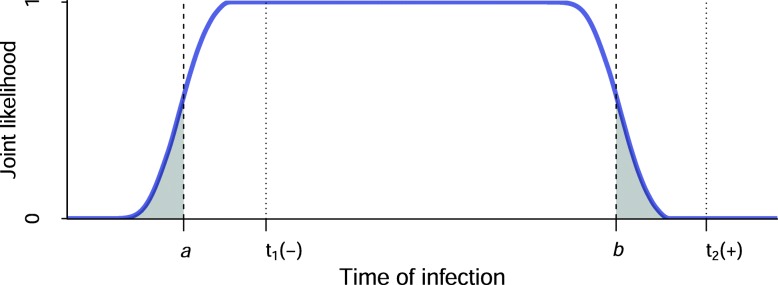
The joint likelihood of obtaining a negative test result at *t*_*1*_ and a positive test at *t*_*2*_, given a hypothetical time of infection. With a uniform prior on time of infection, this can be interpreted as a Bayesian posterior, with the interval [*a,b*] representing the 95% credibility interval

These infection intervals can be understood as plateaus on a very broadly plateaued (rather than ‘peaked’) likelihood function, as shown in Fig. 3. Given a uniform prior, this can be interpreted as a Bayesian posterior, with [*a*, *b*] in Fig. 3 showing the 95% credibility interval (i.e. the interval encompassing 95% of the posterior probability density). Such a posterior, derived from an individual’s diagnostic testing history, could also serve as a prior for further analysis if there is an available quantitative biomarker for which there is a robustly calibrated maturation/growth curve model. We do not deal with this in the present work, but it is explored elsewhere [1], and is an important potential application of this framework and tool.

In Additional file 1: Appendix A we derive a formal likelihood function – i.e. a formula capturing the probability of seeing a data element or set (in this case, the set of negative and positive test results), given hypothetical values of the parameter(s) of interest – here, the time of infection. This interpretation of individual test results relies on the assumption that test results are independent. Of course, the very factors that influence the individual (green) sensitivity curves in Fig. 1 suggest that strong correlations between results of different tests on the same person are likely. Given this, we further demonstrate in Additional file 1: Appendix A when and how test correlation might influence the analysis. While this method does not require a pre-set list of infection stages dependent upon defined assay combinations (as with Fiebig staging), it does require estimation of the diagnostic delay for each assay, either by sourcing direct estimates of the diagnostic delay, or by sourcing such data for a biochemically equivalent assay. Our online HIV infection dating tool, described below, is preloaded with diagnostic delay estimates for over 60 HIV assays, and users can both add new tests and provide alternative diagnostic delay estimates for those tests which are already included.

## Implementation

The public online Infection Dating Tool is available at https://tools.incidence-estimation.org/idt/. The source code for the tool is available publicly under the GNU General Public License Version 3 open source licence at 10.5281/zenodo.1488117. The user-facing web interface is described in Additional file 2: Appendix B.

In practice, the timing of infectious exposure is seldom known, even in intensive studies, and studies of diagnostic test performance therefore provide *relative* times of test conversion [10–12]. Diagnostic delay estimates are therefore anchored to a standard reference event – the first time that a highly-sensitive viral load assay with a detection threshold of 1 RNA copy/mL of plasma would detect an infection. We call this the *Date of Detectable Infection* (DDI). The tool produces a point estimate of this date for each study subject, called the *Estimated Date of Detectable Infection* (EDDI). Details and evaluation of the performance of the diagnostic delay estimates underlying this tool compared with other methods for estimation of infection dates are available elsewhere [1].

The key features of our online tool for HIV infection date estimation are that:

1. Users access the tool through a free website where they can register and maintain a profile which saves their work, making future calculations more efficient.
2. Individual test dates and positive/negative results, i.e. individual-level ‘testing histories’, can be uploaded in a single comma-delimited text file for a group of study subjects.
3. Estimates of the relative ‘diagnostic delay’ between the assays used and the reference viral load assay must be provided, with the option of using a curated database of test properties which provides cited estimates for over 60 HIV assays.
  - If a viral load assay’s detection threshold is known, this can be converted into a diagnostic delay estimate via the exponential growth curve model [1, 2]. We assume that after the viral load reaches 1 RNA copy/mL, viral load increases exponentially during the initial ramp-up phase. The growth rate has been estimated at 0.35 log_10_ RNA copies/mL per day (i.e., a doubling time of slightly less than one day) [2]. The growth rate parameter defaults to this value, but users can supply an alternative estimate.
4. Using the date arithmetic described above, when there is at least one negative test result and at least one positive test result for a subject, the uploaded diagnostic history results in:
  - a point estimate for the date of first detectability of infection (the EDDI);
  - an earliest plausible and latest plausible date of detectable infection (EP-DDI and LP-DDI); and
  - the number of days between the EP-DDI and LP-DDI (i.e., the size of the ‘DDI interval’), which gives the user a sense of the precision of the estimate.

### Access / user profiles

Anyone can register as a user of the tool. The tool saves users’ data files as well as their choices about which diagnostic delay estimates to use for each assay, both of which are only accessible to the user who uploaded them. No personally-identifying information is used or stored within the tool; hence, unless the subject identifiers being used to link diagnostic results can themselves be linked to people (which should be ruled out by pre-processing before upload) there is no sensitive information being stored on the system.

### Uploading diagnostic testing histories

A single data file would be expected to contain a ‘batch’ of multiple subjects’ diagnostic testing histories. Conceptually, this is a table like the fictitious example in Table 1, which records that:

- one subject (Subject A) was seen on 10 January 2017, at which point he had a detectable vial load on an unspecified qualitative viral load assay, but a negative Bio-Rad Geenius™ HIV-1/2 Supplemental Assay (Geenius) result
- another subject (Subject B) was screened negative using a point-of-care (PoC) rapid test (RT) on 13 September 2016, and then, on 4 February 2017, was confirmed positive by Geenius, having also tested positive that day on the PoC RT

**Table:** Table 1

Subject	Date	Test	Result
Subject A	2017-01-10	Qualitative VL	Positive
Subject A	2017-01-10	Geenius	Negative
Subject B	2016-09-13	POC RT	Negative
Subject B	2017-02-04	POC RT	Positive
Subject B	2017-02-04	Geenius	Positive

Sample data file for uploading diagnostic testing histories into the tool

*Abbreviations*: *VL* viral load assay, *Geenius* Bio-Rad Geenius™ HIV-1/2 Supplemental Assay, *POC* point of care, *RT* rapid test

In order to facilitate automated processing, the tool demands a list of column names as the first row in any input file. While extraneous columns are allowed without producing an error, there must be columns named *Subject, Date, Test* and *Result* (not case sensitive). Data in the subject column is expected to be an arbitrary string that uniquely identifies each subject. Dates must be in the standard ISO format (YYYY-MM-DD).

It is fundamental to the simplicity of the algorithm that assay results be either ‘positive’ or ‘negative’. There are a small number of tests, notably Western blot and the Geenius, which sometimes produce ‘indeterminate’ results (partially, but not fully, developed band pattern). Note that there is some lack of standardisation on interpretation of the Western blot, with practice differing in the United States and Europe, for example. While we provide default values for common Western blot assays, users may enter appropriate estimates for the specific products and interpretations in use in their specific context.

We now briefly reconsider Table 1 by adding the minor twist that the Geenius on Subject B is reported as indeterminate. In this case, the data must be recorded as results on either one or both of two separate tests:

1. a ‘Test-Indeterminate’ version of the test – which notes whether a subject will be classified either as negative, or ‘at least’ as indeterminate; and
2. a ‘Test-Full’ version of the test, which determines whether a subject is fully positive or not.

There is then no longer any use for an un-suffixed version of the original test. The data from Table 1 is repeated in Table 2 with differences highlighted. The only changes are the use of the Test-Indeterminate version for Subject A’s negative Geenius result and an indeterminate Geenius result for Subject B. Note that even while Subject A’s test results have not changed, their testing history now looks different, as completely negative results are reported as being negative even for the condition of being indeterminate. Subject B’s indeterminate result on 4 February requires two rows to record, one to report that the test result is not fully negative (positive on ‘Geenius Indeterminate’), and one to report that the result is not fully positive (negative on ‘Geenius Full’). Once diagnostic delays are provided for these two sub-tests, the calculation of infection dates can proceed without any further data manipulation on the part of the user.

**Table:** Table 2

Subject	Date	Test	Result
Subject A	2017-01-10	Qualitative VL	Positive
Subject A	2017-01-10	Geenius Indeterminate	Negative
Subject B	2016-09-13	POC RT	Negative
Subject B	2017-02-04	POC RT	Positive
Subject B	2017-02-04	Geenius Indeterminate	Positive
Subject B	2017-02-04	Geenius Full	Negative

Sample data file for uploading diagnostic testing histories into the tool, with indeterminate results. Abbreviations: VL = viral load assay, Geenius = Bio-Rad Geenius™ HIV-1/2 Supplemental Assay, Full = fully reactive, POC = point of care, RT = rapid test.

### Provision of test diagnostic delay estimates

As described above, tests are summarised by their diagnostic delays. The database supports multiple diagnostic delay estimates for any test, acknowledging that these estimates may be provisional and/or disputed. The basic details identifying a test (i.e. name, test type) are recorded in a ‘tests’ table, and the diagnostic delay estimates are entered as records in a ‘test-properties’ table, which then naturally allows multiple estimates by allowing multiple rows which ‘link’ to a single entry in the tests Table. A test property entry captures the critical parameter of the ‘average’ (usually median) diagnostic delay obtained from experimental data and, when available, a measure of the variability of the diagnostic delay (denoted *σ*).

The system’s user interface always ensures that for each user profile, there is exactly one test property estimate, chosen by the user, for infection dating calculations at any point in time. Users need to ‘map’ the codes occurring in their data files (i.e. the strings in the ‘Test’ column of uploaded data files) to the tests and diagnostic delay estimates in the database, with the option of adding entirely new tests to the database, which will only be visible to the user who uploaded them. The tool developers welcome additional test estimates submitted for inclusion in the system-default tests/estimates.

### Execution of infection date estimation

The command button ‘process’ becomes available when an uploaded testing history has no unmapped test codes. Pressing the button leads to values, per subject, for EP-DDI, LP-DDI, EDDI, and DDI interval, which can be previewed on-screen and downloaded as a comma-delimited file.

By default, the system employs simply the ‘average’ diagnostic delay parameter, in effect placing the EP-DDI and LP-DDI bounds on the DDI interval where the underlying sensitivity curve evaluates to a probability of detection of 0.5. When the size of the inter-test interval (*δ*) is greater than about 20 times the diagnostic delay standard deviation (*σ*), this encompasses more than 95% of the posterior probability.

As an additional option, when values for both *d* and *σ* are available, and a user-specified significance level (*α*), the system will calculate the bounds of a corresponding credibility interval. The bounds of the central 95% (in the case of *α* = 0.05) of the posterior are labelled the EP-DDI and LP-DDI.

### Database Schema

This tool makes use of a relational database, which records information in a set of linked tables, including:

- **subjects:** This table captures each unique study subject, and after infection date estimation has been performed, the subject’s EDDI, EP-DDI, LP-DDI and DDI interval size.
- **diagnostic_test_history:** This table records each test performed, by linking to the subjects table and recording a date, a ‘test code’, and a result. During the estimation procedure, a field containing an ‘adjusted date’ is populated, which records the candidate EP-DDI (in the case of a negative result) or LP-DDI (in the case of a positive result) after the relevant diagnostic delay has been applied to the actual test date.
- **diagnostic_tests:** This is a lookup table listing all known tests applicable to the current purposes (both system-provided and user-provided).
- **test_property_estimates:** This table records diagnostic delay estimates (system and user-provided). It allows multiple estimates per test, with system default estimates flagged.
- **test_property_mapping:** This table records user-specific mapping of test codes by linking each test code in the diagnostic_test_history table to a test in the diagnostic_tests table, as well as the specific test property estimate ‘in use’ by that user for the test in question.

A number of subsidiary tables also exist to manage users of the system and allow linking of personal data files, maps, tests, and test property estimates to specific users.

## Results

### Example of infection date estimates from testing history data

A hypothetical example showing source data and the resulting infection date estimates is provided below. The example data are available with the source code and as Additional file 3 to this article. Table 3 shows the testing history data file, which lists all diagnostic test results obtained for three subjects, which represent typical cases: Subject A had discordant test results on a single date, with the more sensitive test producing a positive result and the less sensitive test a negative result. Subject B seroconverted between two dates separated by some months. Subject C had a large number of tests, and first produced negative results, then discordant results (positive only on a NAT assay), then an immature antibody response, and finally exhibited a fully reactive Western blot. A time series of this kind provides a detailed view of early disease stage progression and yields very precise infection time estimates.

**Table 3:** Example Dataset

Subject	Date	Test	Result
Subject A	2017-01-10	AptimaQualNAT	Positive
Subject A	2017-01-10	GeeniusIndeterminate	Negative
Subject B	2016-09-13	UnigoldRT	Negative
Subject B	2017-02-04	UnigoldRT	Positive
Subject B	2017-02-04	GeeniusFull	Positive
Subject C	2004-10-04	OraQuickRT	Negative
Subject C	2005-11-05	CoulterP24	Negative
Subject C	2010-05-30	GenscreenV2	Negative
Subject C	2014-09-12	AmplicorPooledx10	Positive
Subject C	2014-09-12	BioRadWesternBlotIndeterminate	Negative
Subject C	2014-09-18	ARCHITECT	Positive
Subject C	2014-09-18	BioRadWesternBlotIndeterminate	Positive
Subject C	2014-09-18	BioRadWesternBlotFull	Negative
Subject C	2014-10-04	BioRadWesternBlotFull	Positive

Example dataset for the tool. Abbreviations used in the “Test” column are examples of the type of arbitrary abbreviations a data manager may use to label different diagnostic assays; these abbreviations are defined in the mapping stage, as demonstrated in this case in Table 4.

Table 4 shows the mapping of test codes to tests in the tool’s database, together with median diagnostic delay estimates provided as default estimates in the database.

**Table 4:** Example Mapping

Test code	Database test name	Median diagnostic delay	Ref.
AptimaQualNAT	Aptima HIV-1 RNA Qualitative Assay	4.2	[13]
GeeniusIndeterminate	BioRad Geenius Indeterminate	24.8	[14]
GeeniusFull	BioRad Geenius Fully Reactive	28.8	[14]
UnigoldRT	Trinity Biotech Unigold Rapid HIV Test	25.1	[12]
OraQuickRT-Blood	OraSure OraQuick ADVANCE whole blood	27.7	[12]
CoulterP24	Coulter p24 HIV-1 Antigen Assay	11.5	[2]
GenscreenV2	BioRad Genscreen HIV-1/2 Version 2 Assay	19.1	[15]
AmplicorPooledx10	Pooled Roche Amplicor Monitor v1.5 (ultrasensitive) (Pool of 10)	7.7	[16]
ARCHITECT	Abbott ARCHITECT HIV Ag/Ab Combo	10.8	[12]
BioRadWesternBlotIndeterminate	BioRad GS HIV-1 Western blot Indeterminate	14.8	[10]
BioRadWesternBlotFull	BioRad GS HIV-1 Western blot Fully Reactive	29.6	[12]

Table 5 shows the results of the estimation procedure, together with a column indicating which test results were most informative for deriving the EP-DDIs and LP-DDIs.

**Table 5:** Example Results

Subject	EP-DDI (naïve)	LP-DDI (naïve)	Interval size (naïve)	EP-DDI (95% CI)	LP-DDI (95% CI)	EDDI (95% CI midpoint)	Interval size (95% CI)	*Most informative tests*
Subject A	2016-12-16	2017-01-06	21	2016-12-11	2017-01-05	2016-12-23	25	*GeeniusIndeterminate_Neg 2017-01-10* *AptimaQualNAT_Pos 2017-01-10*
Subject B	2016-08-19	2017-01-06	140	2016-08-21	2017-01-03	2016-10-27	135	*UnigoldRT_Neg 2016-09-13* *GeeniusFull_Pos 2017-02-04*
Subject C	2014-08-28	2014-09-04	7	2014-08-24	2014-09-05	2014-08-30	12	*BioRadWesternBlot-Indeterminate_Neg 2014-09-12* *BioRadWesternBlotFull_Pos 2014-10-04*

Note that the most informative tests are those that exclude the greatest periods of time preceding (in the case of a negative result) and the period following (in the case of a positive result) the earliest dates of plausible detectability, calculated from the test’s diagnostic delay. These are not necessarily the tests performed on the last date on which a negative, or the first date on which a positive result was obtained.

Further note that when the testing interval is small, the 95% credibility interval tends to be wider than the naïve median-based DDI interval (Subjects A and C in the example), but when the testing interval is large, the credibility interval tends to be narrower than the naïve DDI interval (Subject B in the example).

### Use of the tool in real-world research studies

The infection dating tool described in this work has been utilized to estimate infection dates for all subjects who contributed specimens to the CEPHIA repository, where diagnostic testing histories could be obtained. A key aspect of that consortium’s work has been to characterise tests for recent HIV infection (HIV ‘incidence assays’) – in particular by estimating the two critical performance characteristics, the mean duration of recent infection (MDRI) and false-recent rate (FRR), which would not have been possible without individual-level infection time estimates (see, for example, [8]).

## Discussion

The whole code base for the tool is available in a public source code repository [17], and so anyone can deploy their own copy of the tool, or ‘fork’ the repository (i.e. make their own copy of the code repository) and make any modifications they wish. The only condition is that the origin of the code is acknowledged, and that dissemination of the modified code is also in open source form under the same licensing. The developers of the tool welcome contributions to the code, which can be proposed through ‘pull requests’ issued on the source code hosting platform. Test characteristics for more than 60 common HIV diagnostic tests are included in the code base and are easy to update as new data become available.

Consistent infection dating could be of interest in the study of other infections. Only minor modifications and a database of tests and test property estimates would be required to deploy a separate version of the system to handle other infections. This would be especially useful in contexts where multiple diagnostic platforms or algorithms have been used within a single dataset intended for a unified analysis.

Even in intensive studies from which ‘diagnostic delay’ estimates are drawn, it is rarely possible to determine the actual date of infectious exposure. We have adopted a nomenclature based on the earliest date on which an infection would have had 50% probability of being detected, using a viral load assay with a detection threshold of 1 copy per ml, and we refer to this as the Date of Detectable Infection (DDI).

Consistent dating of infection events across subjects has obvious utility when analysing multi-site datasets that contain different underlying screening algorithms. Consistent use of ‘diagnostic history’ information is also valuable for individual-level interpretation of infection staging at diagnosis. However, a limitation of this approach is that it relies on details of diagnostic testing histories that are often not recorded or clearly reported. For example, it may be noted that a subject produced a negative Western blot result on a particular date, but without recording of the specific product and the interpretive criteria employed. This challenge is further compounded by country-specific variations in assay names and interpretive criteria for the same assays.

There are two cases in which this method cannot be employed. First, if the first HIV test an individual ever has is a fully reactive test (i.e. no negative test result is ever reported for that individual, on any assay), there is no way to create an infection time interval. Luckily, given WHO 90–90-90 targets and PEPFAR testing programs throughout the world, it has become increasingly common for individuals to test for HIV regularly. Second, self-reported testing histories may lack precise information on the dates of tests and the specific assays used, in which case this tool cannot be used to estimate infection time. If a “likely” assay can be determined (e.g. by substituting with a typical country testing algorithm) this can serve as a proxy, with some unknown level of bias introduced into the estimate. Lastly, when a last negative result and a first positive result are separated by a long period of time, very uninformative infection time estimates are produced by this method. In these cases, the interpretation of additional quantitative markers – utilising the infection time intervals estimated by this tool as ‘priors’ – can yield informative estimates [1].

A simple method for interpreting additional quantitative markers (such as a signal-to-cut-off ratio from the ARCHITECT diagnostic assay or a normalised optical density from the Limiting Antigen Avidity recency assay) is to report the Mean Duration of Recent Infection associated with an equivalent recency discrimination threshold as a ‘time scale’: on average, a subject producing *y* quantitative result has been infected for less than *x* days – see for example [8].

## Conclusions

In many settings, including most research studies, detailed diagnostic testing data are routinely recorded, and especially when regular testing occurred, can provide reasonably precise estimates of the timing of HIV infection from purely qualitative results.

We have presented a simple logic for the interpretation of ‘diagnostic testing histories’ into ‘infection time estimates’ as a point estimate (EDDI) and an interval (EP-DDI – LP-DDI), implemented in a publicly-accessible online tool that supports wide application of this logic.

## Availability and requirements

**Project name:** Infection Dating Tool

**Project home page:** https://tools.incidence-estimation.org/idt/

**Source code:** https://github.com/SACEMA/infection-dating-tool/

**Latest release:** 10.5281/zenodo.1488117

**Operating systems:** Platform independent

**Programming language:** Python

**Other requirements:** Python 2.7.x, Django 1.9.6

**License:** GNU GPL-3

## Supplementary information

**Additional file 1: Appendix A.** Formal likelihood function and impact of test correlation. (PDF 949 kb)

**Additional file 2: Appendix B.** Infection Dating Tool Web Interface. (PDF 963 kb)

**Additional file 3:** Example Testing History Dataset. A dataset of the form that can be processed by the Infection Dating Tool, for three subjects. The data in this file are demonstrative and do not come from real subjects. Subject A had discordant test results on a single date, with the more sensitive test producing a positive result and the less sensitive test a negative result. Subject B seroconverted between two dates separated by some months. Subject C had a large number of tests, and first produced negative results, then discordant results (positive only on a NAT assay), then an immature antibody response, and finally exhibited a fully reactive Western blot. (CSV 704 bytes)

## Data Availability

The example dataset analysed during this study is published in its full form in this article and also available with the source code of the tool. All source code is available from a public repository under an open source license, using the persistent DOI: 10.5281/zenodo.1488117. Other datasets analysed using this tool, including CEPHIA data, are not publicly available, since they contain personally identifying information, notably actual dates of HIV test results. Anonymised data with modified dates can be obtained from the corresponding author upon reasonable request.

## Abbreviations

CEPHIA: Consortium for the Evaluation and Performance of HIV Incidence Assays
CI: Credibility Interval
DDI: Date of Detectable Infection
EDDI: Estimated Date of Detectable Infection
EP-DDI: Earliest Plausible Date of Detectible Infection
HIV: Human Immunodeficiency Virus
LP-DDI: Latest Plausible Date of Detectable Infection
PoC: Point-of-Care
RNA: Ribonucleic Acid
RT: Rapid Test
