# Supplementary material for: Interpreting HIV diagnostic histories into infection time estimates: analytical framework and online tool
Source: BMC Infect Dis. 2019 Oct 26;19:894. doi: 10.1186/s12879-019-4543-9 (PMC6815418; doi:10.1186/s12879-019-4543-9)

Appendix A: Formal likelihood function and impact of test correlation

It is analytically useful to specify an explicit 'likelihood function', i.e. a formula for capturing the probability of seeing a data element (or set), given some hypothetical values for parameters which determine the behaviour of the underlying system, including the measurement process. This facilitates all the usual statistical manipulations for obtaining confidence intervals, Bayesian posteriors, etc. For the present application, test sensitivity curves such as those in Figure 1 in the main mansuscriptare precisely the likelihood of obtaining a positive result upon application of a given test, at a given time since infection. The likelihood of obtaining a negative result, on this very application of the test, is simply 1 minus the likelihood of obtaining a positive result, i.e. a vertically flipped version of the test sensitivity curve. As noted above, meaningful infection dating relies on having at least one negative test result and at least one positive test result.

Classical test conversion series: To begin, we consider precisely one negative and one positive test result, arising from two different study interactions by a single subject, at times t_1 and t_2 respectively, separated by some duration δ. In order to make inferences about the time of infection, we construct a likelihood function which expresses the probability of seeing these two particular results, *as a function of a hypothetical infection time*. This kind of likelihood (of two observations) is usually written as the product of:

- the likelihood of seeing one result (chosen arbitrarily to be considered first) given the hypothetical time of infection, and
- the likelihood of seeing the other result, given
  - the same hypothetical time of infection, and
  - the fact that the other result has in fact been obtained.

Using T_{inf} to denote the actual time of infection that we are trying to estimate, t_{inf} to denote particular values of hypothetical infection time and $[+, t_n]$ and $[-, t_n]$ to denote positive and negative test results at observation times t_n, respectively, this can be written as:

$$\begin{aligned} L(t) &\equiv L\big([-, t_1], [+, t_2] \big| T_{inf} = t\big) \\ &= L\big([-, t_1] \big| T_{inf} = t\big) \cdot L\big([+, t_2] \big| \{[-, t_1], T_{inf} = t\}\big) \\ &= L\big([+, t_2] \big| T_{inf} = t\big) \cdot L\big([-, t_1] \big| \{[+, t_2], T_{inf} = t\}\big) \end{aligned}$$

capturing that the likelihood of seeing both of two events (A and B) is equal to either

1. *the likelihood of A* multiplied by *the likelihood of B, given A*, i.e. $L(A) \cdot L(B|A)$, or
2. *the likelihood of B* multiplied by *the likelihood of A, given B*, i.e. $L(B) \cdot L(A|B)$.

The details of the *conditioned* likelihoods, which might be complex, must necessarily be such that the two formulations are equivalent. We will focus in detail on the first formulation, as it seems more intuitively appealing when $t_1 < t_2$.

Figure A.1. shows, in thick green and red, respectively, the *population-level* likelihoods of observing the negative test result at t_1 and the positive test result at t_2. A subset of the family of *individual-level* curves, chosen to visually suggest their distribution, is indicated as thin lines. A close look at these curves reveals that they are the horizontally flipped (and in the case of the green curves, also vertically flipped) test sensitivity curves of the tests performed (compare with the detailed shapes in Figure 1 of the main manuscript). These curves display information for each test result, considered independently.

Figure A.1.

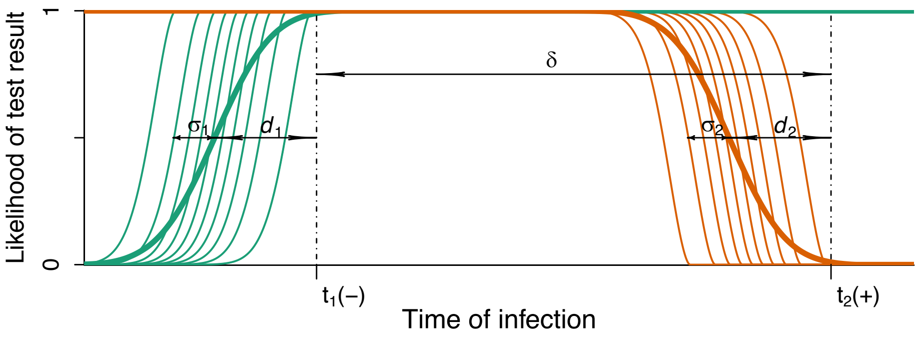

The fundamental point of estimating an infection time is that both tests were in fact performed on the same individual. It is highly likely that those individuals who convert rapidly, post infection, on $Test_1$ also convert rapidly on $Test_2$ – which might, after all, be the same test, and is likely to be a similar test. The details of this conditioning can in principle be complex, and it is infeasible to study all the correlations between all tests in use in studies. A critical question, then, is whether, when, and how this correlation impacts the conditioned likelihoods which are the fundamental building block of a forma inference of infection time from diagnostic testing histories.

The 'worst case' scenario would be when the correlation is very strong, as it would be if the tests performed at the two times are in fact the same test. We have explicitly implemented a model of test sensitivity based on the following points:

- the performance of any test is defined by a family of N individual-level sensitivity curves of the type in Figures 1 (main manuscript) and A.1.
- for a particular test, each individual-level curve is a shifted Weibull with the same shape and scale parameter.
- The shift parameter is normally distributed, though with a discretised realisation, with step $\epsilon = \frac{1}{N}$ – i.e. we assign individual diagnostic delays (Weibull shift parameters) to the percentiles $\frac{1}{2}\epsilon, \frac{3}{2}\epsilon, \ldots, 1 - \frac{1}{2}\epsilon$ of a normal distribution.
- The mean of the distribution of shift parameters is a test's mean diagnostic delay (d in Figures 1 (main manuscript) and A.1.), and the standard deviation (σ in Figures 1 and A.1.) manifests as something akin to a shape parameter of the population-level curve.

To keep the scenario simple initially, we first consider the case when the two test times differ by more than $d + \sigma$. We later consider the complication of the other extreme, i.e. when the positive and negative test results are obtained on the same day, and the distributions of the diagnostic delays overlap substantially.

The behaviour of the fully-conditioned likelihood expression

$$L\big([-, t_1], [+, t_2]\big|T_{inf} = t\big) = L\big([-, t_1]\big|T_{inf} = t\big) \cdot L\big([+, t_2]\big|\{[-, t_1], T_{inf} = t\}\big)$$

can then be understood by considering how the factor $L\big([+, t_2]\big|\{[-, t_1], T_{inf} = t\}\big)$ might differ from the naïve population-averaged, unconditioned $L\big([+, t_2]\big|T_{inf} = t\big)$. The latter is what one can obtain from a study investigating the performance of one or several diagnostic tests, without having to apply the particular test combinations to particular individuals.

We now analyse the various ranges of t_{inf} which are qualitatively different from each other:

Values of t_{inf} *on the far-left end of the timeline:* For very 'early' hypothetical infection times, the likelihood of seeing the negative result at t_1 becomes very small. If that negative result has indeed occurred at t_1, it would normally be the result of a laboratory error, which would have a reasonable chance of being detected with strong quality controls. If the error remains undetected, testing positive at t_2 (a time later than t_1 by a significant margin) is nevertheless almost assured, i.e.

$$L\big([+,t_2]\big|\{[-,t_1],T_{inf}=t\}\big) \approx L\big([+,t_2]\big|T_{inf}=t\big) \approx 1$$

So, in this case there is no discernible difference between the unconditioned and conditioned likelihood, although there is no analytical cure for a false negative result.

Values of t_{inf} *within the dynamic range of the* $Test_1$ *sensitivity curve*: Figures A.2.a-A.2.e consider a series of hypothetical infection times (t_{inf}) that span the likely range of diagnostic delays.

Figure A.2.a, indicating that the negative result at t_1 occurs somewhat longer after infection than the mean diagnostic delay, is suggestive of the subject being a significantly slower-than-average progressor on the diagnostic marker. This is captured by the dotted green (faster) individual progression curves for $Test_1$, indicating their reduced plausibility. Correspondingly, only the slowest progression rates are plausible among the red curves for $Test_2$. Nevertheless, given the location of t_{inf}, namely long before the application of $Test_2$, it does not matter which of the $Test_2$ progression curves the individual is likely to be on – they all evaluate to 1 so long after infection.

Figure A.2.b, indicating that the negative result at t_1 occurs at a time after infection approximately equal to the mean diagnostic delay, is suggestive of the subject not being a significantly faster-than-average progressor on the diagnostic marker. This is captured by the reduced number of dotted green (fastest) individual progression curves for $Test_1$. Correspondingly, the fastest progression rates are less plausible among the red curves for $Test_2$. Once more, given the location of the hypothetical infection time, namely long before the application of $Test_2$, it does not matter which of the $Test_2$ progression curves the individual is likely to be on – they all evaluate to 1 so long after infection.

Figure A.2.c, indicating that the negative result at t_1 occurs at a time after infection that is significantly less than the mean diagnostic delay, is consistent with all but one of the green and hence red individual progression lines are plausible. Not only does the negative result at t_1 not imply significant conditioning on the subject's diagnostic marker progression rate, but all the individual-level red curves in any case evaluate to 1 *at the time of* $Test_2$.

Values of t_{inf} *anywhere near, or to the right of* t_1: For these 'later' hypothetical infection times, we *expect* to see a negative result for the test at t_1, even more so than in Figure A.2.c, and so, the negative result provides no information on the question of whether the subject is prone to rapid or slow test conversion. Hence, no modification is implied of $L\big([+,t_2]\big|\{[-,t_1],T_{inf}=t\}\big)$ relative to the population average $L\big([+,t_2]\big|T_{inf}=t\big)$, though of course in this region there are many values of t_{inf} for which this likelihood is not approximately 1. Figures A.2.d and A.2.e show values of t_{inf} on the 'plateau' and on the 'descent' from the plateau in the dynamic range of diagnostic delays of $Test_2$.

Figure A.2.

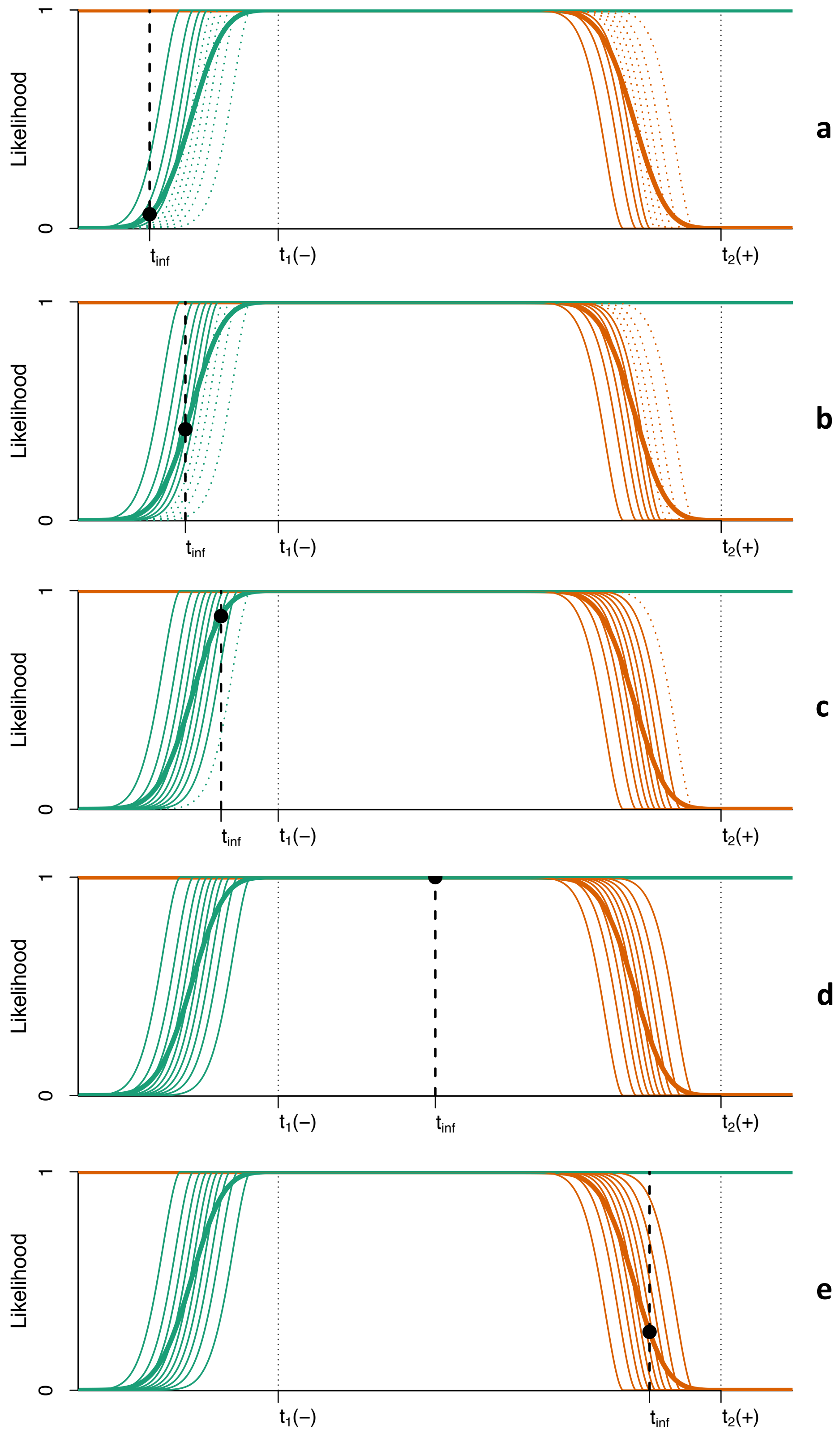

The three zones of t_{inf} discussed above account for the full range of values of t for which the joint likelihood is to be constructed. It is clear that the full joint likelihood is indeed given by the product of the unconditioned population-level likelihoods for the two test results, as shown in Figure A.3. As the curves obtain values indistinguishable from either 0 or 1 for much of their range, this product is little more than a superposition of the two curves.

Figure A.3.

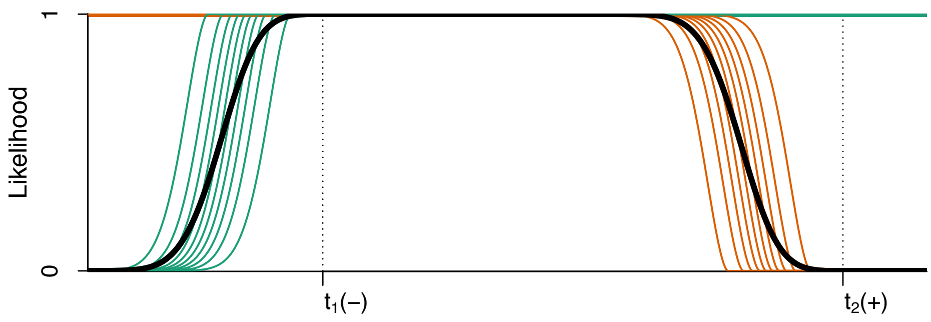

Figure 3, shown in the main manuscript, indicates where this round-shouldered plateau is located relative to the test dates and population-averaged diagnostic delays. Under the parametric assumptions outlined above, one may specify a 'confidence level' (such as usually encapsulated in a significance level α, chosen to be 0.05 in Figure 3) and calculate the bounds of the (in our case, 95%) 'credibility interval' $[a, b]$, encompassing the relevant proportion of the posterior probability density $p(t)$. We therefore find the values of a and b that satisfy

$$\int_{-\infty}^{a} p(t)\, dt = \int_{b}^{\infty} p(t)\, dt = \frac{\alpha}{2}$$

Note that when $t_1(-)$ and $t_1(+)$ are separated by a substantial period of time, the credibility interval is likely to be narrower than naïve bounds defined simply by the population-average diagnostic delays. When the period is short, the credibility bounds are likely to shift outward from the naïve bounds.

Discordant results on a given study-visit: Figure A.4. shows the typical 'discordant test' situation, where a test with a longer diagnostic delay produces a negative result and a test with a shorter diagnostic delay produces a positive result, at the same visit.

Figure A.4.

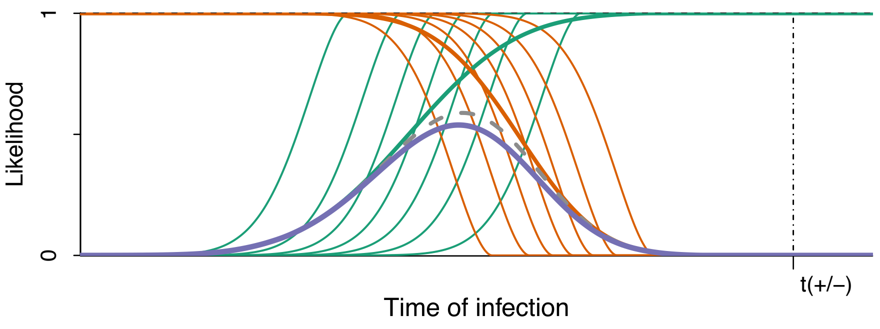

Even here, though not as starkly as in the case where the two tests are conducted at significantly different times, conditioning one result on the other has relatively modest impact. Moving the hypothetical infection time to the left, the negative result becomes less likely, and the effect of the conditioning on the

likelihood of seeing the second test result becomes more significant. However, as the hypothetical infection time moves further left, the times under consideration leave the dynamic range of the positive test, and it becomes ever less plausible that a negative test result is obtained. We do not explicitly display figures indicating the conditioning implied for various hypothetical values of infection time, but merely indicate in the solid blue curve the formally calculated fully specified joint likelihood which takes this conditioning into account in terms of the extreme correlation model outlined above. This exact likelihood does not differ meaningfully from the simple product of the population-level likelihoods of the two tests (shown dashed, in grey). The main conclusion, then, is that relative to the test date, plausible infection times are largely located between the two diagnostic delays (with some spreading due to variability).

Figure A.5. shows the situation where the dynamic ranges of the tests are essentially the same. In this case, the plausible dates of infection are centred around the shared diagnostic delay of the tests, again with some spread for variability. The relatively small amplitude of the exact curve indicates that the fully conditioned discordancy is significantly less likely to occur than one would infer from a naïve calculation, but the key point is that the infection time estimate is not affected at the level at which it can be plausibly reported.

Figure A.5.

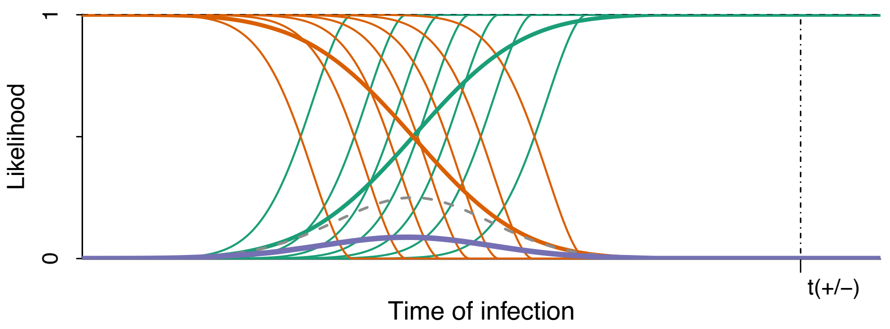

Figure A.6. shows an outlier situation in which a more sensitive test is negative while a less sensitive test is positive. Relative to the naïve product of likelihoods, the correctly specified joint likelihood is very small for all values of *t*. This indicates that such anomalous discordant results are extremely rare, arising most plausibly from test error. If such an outlier occurs without test error, the fully-conditioned likelihood could differ significantly from the naïve one; however, it would depend on essentially unknowable details of distributional tails and test correlation, and such cases are sufficiently rare to have no impact on conclusions drawn from observing large numbers of individuals. Note that the extreme rarity of anomalous discordant results is a function of the very strong intra-test correlation assumed in this model; in reality the intra-test correlation is likely far less strong, making these events less rare but also lessening the discrepancy between the naïve and fully-conditioned likelihood.

Figure A.6.

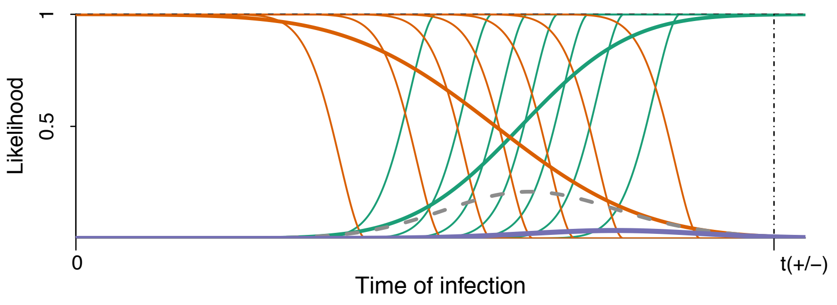

Supplement: Supplementary file 1 — Additional file 1: Appendix A. Formal likelihood function and impact of test correlation. (PDF 949 kb) [file 12879_2019_4543_MOESM1_ESM.pdf]
