# Supplementary material for: Interpreting HIV diagnostic histories into infection time estimates: analytical framework and online tool
Source: BMC Infect Dis. 2019 Oct 26;19:894. doi: 10.1186/s12879-019-4543-9 (PMC6815418; doi:10.1186/s12879-019-4543-9)

Appendix B: Infection Dating Tool Web Interface

Supplementary material to Grebe E, Facente SN, Bingham J, Pilcher CD, Powrie A, Gerber J, Priede G, Chibawara T, Busch MP, Murphy G, Kassanjee R, Welte A. Interpreting HIV Diagnostic Histories into Infection Time Estimates: Analytical Framework and Online Tool.

Once logged in, the system presents users with four primary pages, accessible via links spread in horizontal tabs below the header, as shown in Figure 1. The first three are described in turn below, with the fourth the subject of a separate publication (Welte, et al., forthcoming).

Figure B.1: Navigation[a]

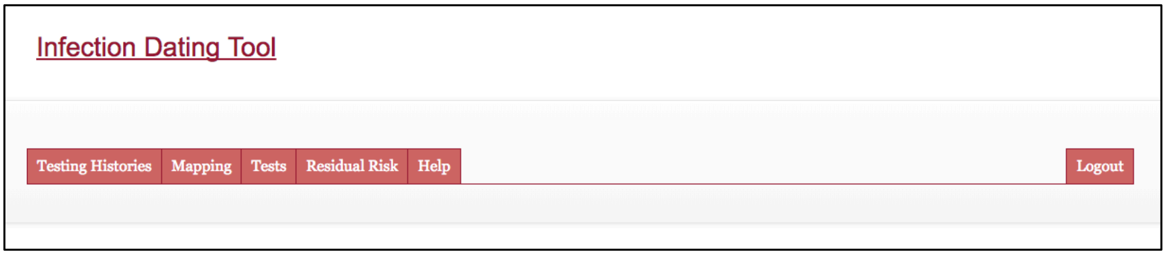

[a]*The logic and diagnostic test performance data required for infection dating has significant overlap with that required to calculate the residual risk of infectious material being missed by screening algorithms applied to blood products. Therefore, the online tool has a residual risk calculator built into it as well. This aspect of the tool is discussed and presented in a separate article (Welte et al., forthcoming).*

Testing Histories

This tab (Figure 2) allows users to locate, view and delete previously uploaded 'testing histories', and to upload new ones. It is also where users trigger the action of processing the uploaded testing histories into 'infection dating estimates', which can then be viewed and downloaded.

Figure B.2: Testing Histories

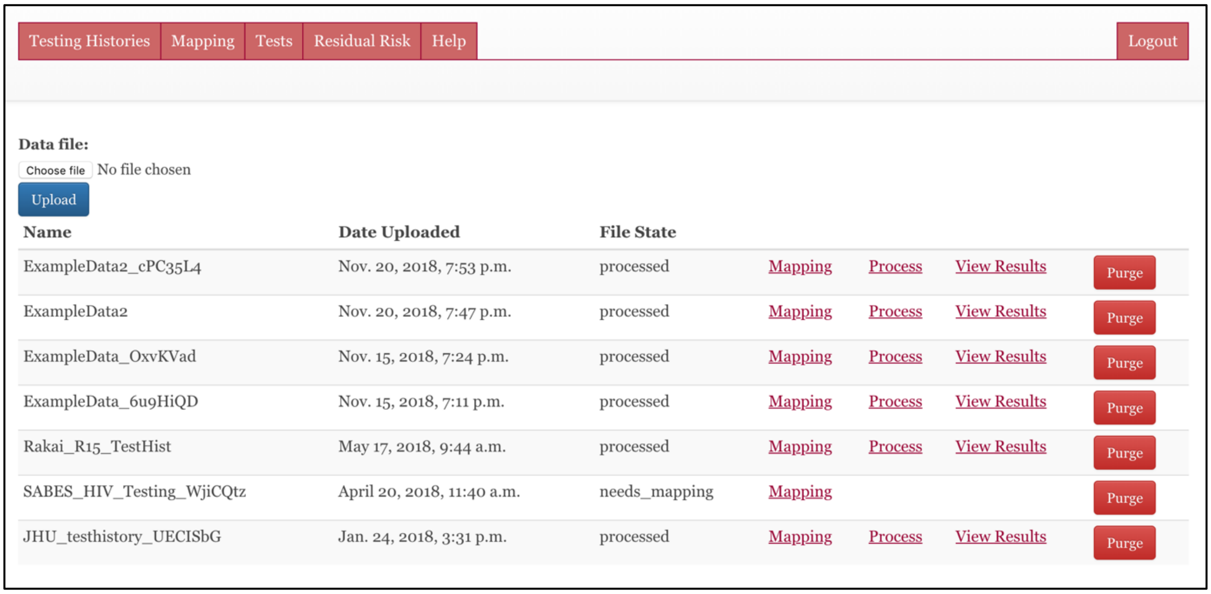

Mapping

This tab (Figure 3) allows users to link strings (alphanumeric codes) in their data files to tests in the online database, hence linking records in uploaded files to the applicable diagnostic delays.

Figure B.3: Mapping

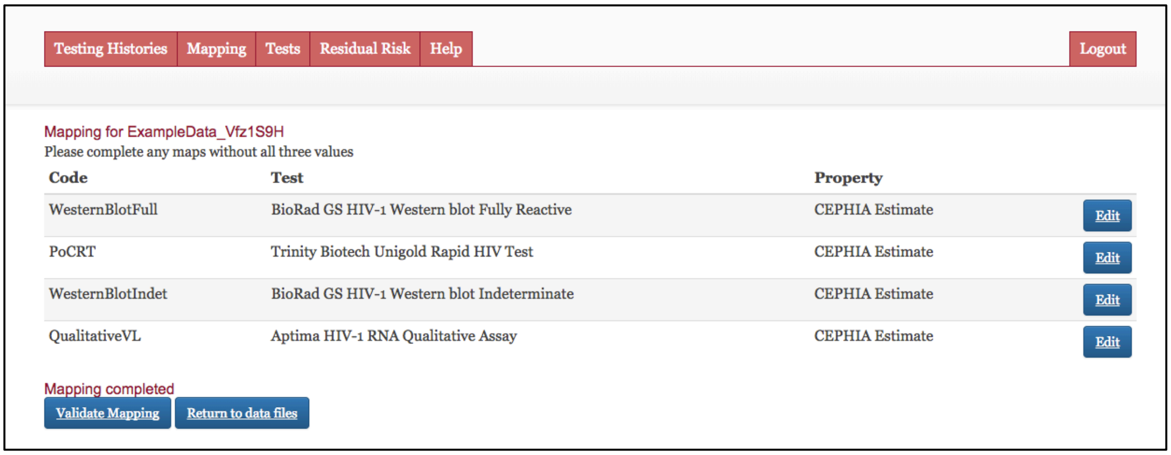

Tests

This tab (Figure B.4a) allows users to view the existing database of diagnostic tests, and to add new ones if necessary. Note that each user sees only the shared developer-maintained list of tests, plus his/her own – not those added by other users. This page further allows the user to select between computing EP-DDI and LP-DDI using naïve diagnostic delay medians, or to utilise the σ parameter and a specified value of α to compute credibility intervals (see Figures B.4b and B.4c).

Figure B.4a: Tests

Calculation parameters: *EP- & LP-DDI will represent 95% Credibility Intervals*

Your tests

Add a new test

Name	Category	
AmplicorIndDBS1000	Viral Load	Edit
Ampliscreen pool of 5	Viral Load	Edit
BioMerieux Vitek WB Fully Reactive	Western blot	Edit
BioMerieux Vitek WB Indeterminate	Western blot	Edit
Generic Viral Load DT42	Viral Load	Edit
ID-NAT ULTRIO	Viral Load	Edit
MP-NAT (pool of 16, LoD50 of 1.7)	Viral Load	Edit
MP-NAT (pool of 8, LoD50 of 1.7)	Viral Load	Edit
PooledNATDT100x5 (effective 500)	Viral Load	Edit
SANBS NAT	Viral Load	Edit

Global tests

Western blot

BioRad GS HIV-1 Western blot Fully Reactive	Edit
BioRad GS HIV-1 Western blot Indeterminate	Edit

1st Gen Lab Assay (Viral Lysate IgG sensitive Antibody)

Murex ICE HIV-1.O.2 EIA	Edit
Unspecified 1st Gen Lab Assay	Edit

Figure B.4b: Naïve estimates using median diagnostic delays

Calculation parameters: *EP- & LP-DDI will be based on Median diagnostic delays*

Viral load growth rate estimate

0.35

Fiebig et al. (AIDS 2003): 0.35 $\log_{10}$ copies/ml/day

Calculate DDI bounds:

Using Median diagnostic delays

Reset to defaults | Submit

Figure B.4c: Computing credibility intervals

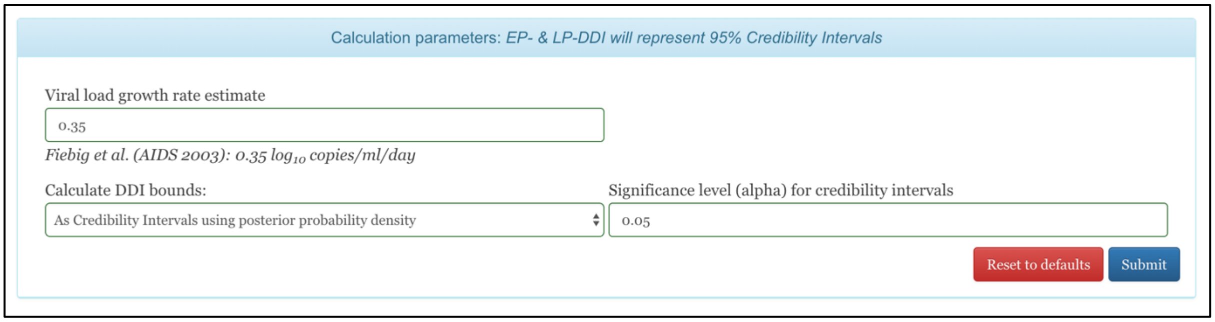

Results

Processing can be triggered after test codes have been mapped to specific assays in the database. If test property estimates other than the default are preferred, these can be selected on the mapping screen prior to processing. Each file that has been uploaded on the "Testing Histories" tab has a "Mapping" link, and once mapping has been completed, a "Process" link appears. After processing, results can be viewed and downloaded on a per-file basis. Figure B.5a shows EP-DDI and LP-DDI based on median diagnostic delays, and Figure B.5b shows 95% credibility intervals.

Figure B.5a: Results

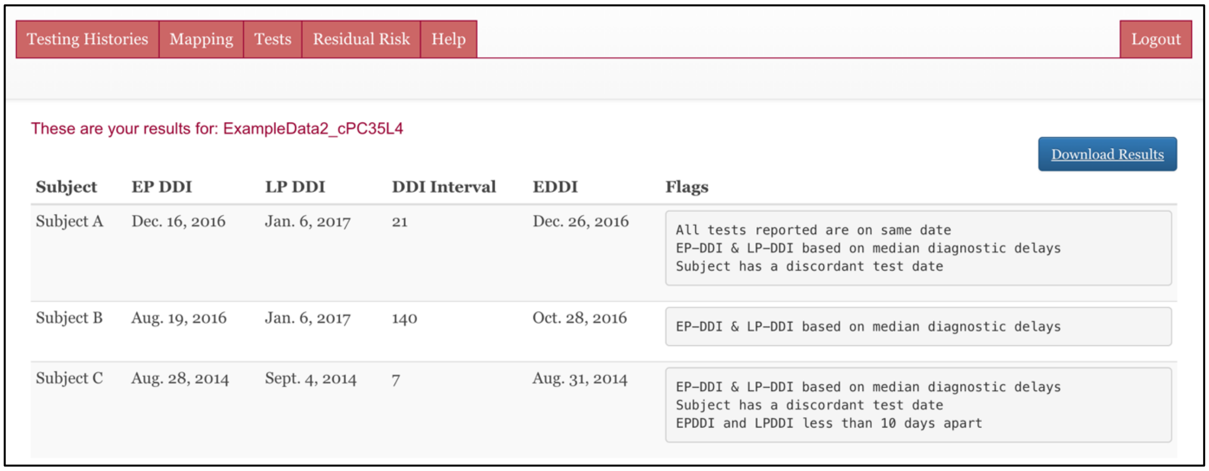

Figure B.5b: Results (95% CIs)

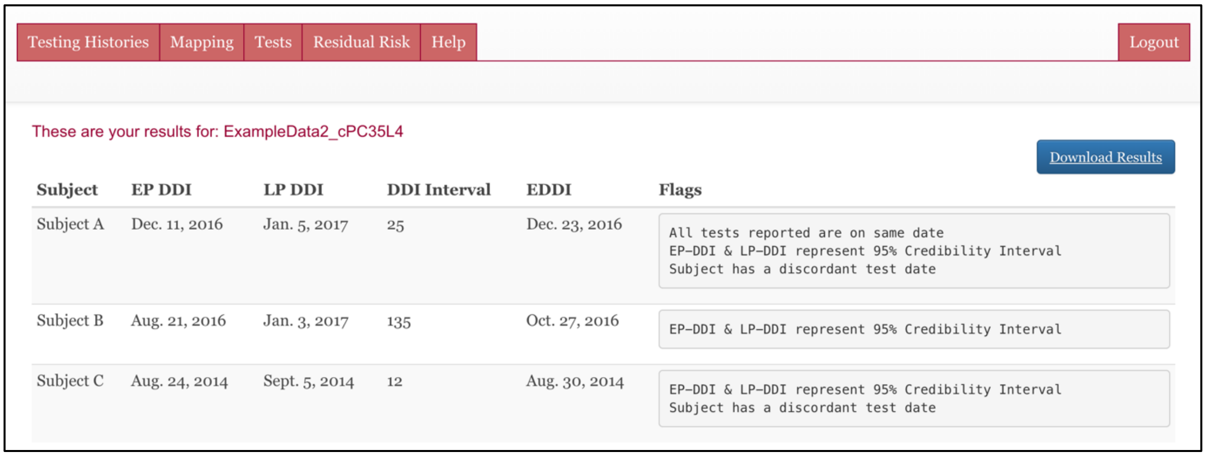

Supplement: Supplementary file 2 — Additional file 2: Appendix B. Infection Dating Tool Web Interface. (PDF 963 kb) [file 12879_2019_4543_MOESM2_ESM.pdf]
